# Identification of the Prognostic Value Among Suppressor Of Cytokine Signaling Family Members in Kidney Renal Clear Cell Carcinoma

**DOI:** 10.3389/fmolb.2021.585000

**Published:** 2021-12-02

**Authors:** Changjiu Li, Wenhao Zhang, Tiantian Fang, Ning Li, Yuwei Wang, Lugeng He, Huadong He

**Affiliations:** ^1^ Department of Urology, Affiliated Hangzhou First People’s Hospital, Nanjing Medical University, Hangzhou, China; ^2^ Department of Urology, Zhejiang Chinese Medical University, Hangzhou, China; ^3^ Department of Urology, Affiliated Hangzhou First People’s Hospital, School of Medicine, Zhejiang University, Hangzhou, China

**Keywords:** Suppressor of cytokine signaling, prognosis, biomarker, bioinformatics analysis, renal clear cell carcinoma

## Abstract

**Background:** Kidney renal clear cell carcinoma (KIRC) has become one of the most prevalent malignancies worldwide and remains a crucial cause of cancer-related morbidity and mortality. Aberrant activation of the JAK/STAT pathway acts as an important role in KIRC. The suppressor of cytokine signaling (SOCS) family members are the key negative regulators of the JAK/STAT pathway. SOCS family members have been verified to act as significant roles in regulating cellular responses to many cytokines and growth factors. However, whether the expression levels of SOCS affect the prognosis of patients with KIRC is still elusive.

**Methods:** We first evaluated the expression of SOCS family genes in KIRC and determined the correlation between SOCS expression and different clinicopathological features. Then, we analyzed the genetic alterations, potential functions, transcription factor targets, and immune infiltration of SOCS family members based on the information available on public databases. Finally, we assessed the prognostic value of differentially expressed SOCS family members.

**Results:** The expression levels of *SOCS2*, *SOCS4*, *SOCS6*, *SOCS7*, and *CISH* were downregulated in KIRC, and all SOCS genes were associated with clinicopathological features of patients with KIRC. SOCS family members have been predominantly related to protein binding, signaling adaptor activity, and JAK/STAT cascade. We found that STAT3, STAT6, and IRF1 are the key transcription factors that may be participated in the regulation of SOCS. We also found an association between the expression levels of SOCS and the immune infiltrates of KIRC. Finally, we have illuminated that *SOCS1* and *SOCS3* are risky genes, whereas *SOCS2*, *SOCS4*, *SOCS6*, *SOCS7*, and *CISH* are some of the protective genes for patients with KIRC; based on these, we have created a KIRC prognostic index for predicting the prognosis of patients of KIRC.

**Conclusion:** Our study may contribute to further understanding the functions of SOCS genes in KIRC, which may help clinicians in selecting the appropriate drugs and predicting the outcomes for patients with KIRC.

## Introduction

Kidney cancer has become the 16th most prevalent malignancy worldwide, representing 2.2% of all new cancer cases. Nearly 403,000 new patients and 175,000 related deaths were reported in 2018 worldwide (Bray et al., 2018). Renal cell carcinoma (RCC) is the most prevalent malignancy of renal parenchyma in the urinary system and is responsible for up to 85% of the cases (Barata and Rini, 2017). The kidney renal clear cell carcinoma (KIRC) is the most prevalent histological subtype of RCC. In recent years, traditional surgery, radiotherapy, chemotherapy, and immunotherapy have already been used for the treatment of patients with KIRC (Barata and Rini, 2017). However, the morbidity and mortality of KIRC patients remain high. Currently, tumor–nodes–metastases stage, histological grade, and necrosis are regarded as independent predictors of prognosis for patients with KIRC (Zigeuner et al., 2010). Nevertheless, patients with the same clinicopathological characteristics often have different outcomes. Therefore, further research is warranted to acquire a deeper insight into the mechanism of KIRC and identify biomarkers for the diagnosis and prognosis of KIRC.

Aberrant activation of JAK/STAT acts as an important role in KIRC, and the suppressor of cytokine signaling (SOCS) family members are the key negative regulators of the JAK/STAT pathway (Horiguchi et al., 2002a; Horiguchi et al., 2002b; Linossi and Nicholson, 2015; Santoni et al., 2015). They were first discovered in the 1990s (Yoshimura et al., 1995; Starr et al., 1997) and were later verified to act as critical roles in managing cellular responses to many cytokines and growth factors (Linossi and Nicholson, 2015). The SOCS family is comprised of eight members, including cytokine-inducible SH2-containing protein (*CISH*) and *SOCS1-7*, which are composed of a central SH2 domain and a C-terminal SOCS box motif (Hilton et al., 1998). Functionally, *SOCS1-3* and *CISH* are involved in the negative regulation of the JAK/STAT pathway, which is abnormally activated during tumorigenesis and malignant progression in multiple cancers (Gao et al., 2013a; Li et al., 2019; Ren et al., 2019). *SOCS1* and *SOCS3* have the unique ability to directly manage the enzymatic activity of JAK kinase. However, there are fewer studies on SOCS4-7; they may act as an important role in multiple protein targets, but the specific functions of SOCS4-7 remain elusive. Zhang et al. (2019) found that *SOCS5* could promote metastasis of hepatocellular carcinoma *via* the activation of the PI3K/Akt/mTOR pathway. *SOCS7* may suppress the development of prostate cancer *via* the activation of the JAK/STAT pathway (Ge et al., 2012). As important negative regulators of the JAK/STAT signaling pathway, there is evidence to suggest that SOCS family genes could potentially act as critical roles in the development of several human diseases, including several types of cancers (Quentmeier et al., 2008; Ge et al., 2012; Gao et al., 2013a; Li et al., 2019; Ren et al., 2019).

Previous studies have revealed the functions of several SOCS family genes and JAK/STAT pathway in kidney cancer (Horiguchi et al., 2002c; Stofas et al., 2014; Wake and Watson, 2015; Yabe et al., 2018; Liang et al., 2020b); however, whether the expression levels of SOCS can affect the prognosis of patients with KIRC is not fully understood. Recently, with the development of unique prediction techniques, it is possible to fully understand the role of SOCS family members. In this study, we carried out a comprehensive analysis of the transcriptional levels of SOCS family members in KIRC. We also assessed the values of these SOCS genes as prognostic biomarkers, thereby providing a reliable foundation for evaluating the prognosis of patients with KIRC and selecting suitable treatment options. This research may be helpful to further the comprehending of the functions of the SOCS family of genes in KIRC, helping clinicians select the appropriate drug and predict the outcomes for patients with KIRC.

## Materials and Methods

### The Cancer Genome Atlas Data

Normalized RNA-seq data and relevant clinical data for KIRC samples (*n* = 539) and normal renal tissues (*n* = 72) were obtained from The Cancer Genome Atlas (TCGA) (https://portal.gdc.cancer.gov/) database on June 9, 2020. For messenger RNA (mRNA) expression data, the HTSeq-FPKM format was obtained from TCGA. Table 1 shows the clinicopathological details of 539 KIRC patients.

**TABLE 1:** Clinicopathological details of 539 KIRC patients.

		Cases
Age	<65	337
	≥65	202
Sex	Famale	191
	Male	348
T stage	T1	276
	T2	69
	T3	183
	T4	11
N stage	N0	241
	N1	17
	Nx	281
M stage	M0	428
	M1	79
	Mx	32
Stage	Ⅰ	270
	Ⅱ	57
	Ⅲ	126
	Ⅳ	83
	unknow	3
Grade	G1	14
	G2	231
	G3	208
	G4	78
	Gx	8

### UALCAN

UALCAN (http://ualcan.path.uab.edu) is a user-friendly and interactive web portal that provides analyses of gene expression data based on TCGA database (Chandrashekar et al., 2017). We analyzed the expression levels of SOCS family members in KIRC and normal renal tissues based on the TCGA analysis module of the UALCAN database. In this study, a *p*-value of 0.05 was set as a threshold.

### cBioportal

cBioPortal (http://cbioportal.org) is a comprehensive and interactive web portal that creates a user-friendly platform to assess multidimensional cancer genomics data, including mRNA expression levels and mutation data (Gao et al., 2013b). We analyzed the genetic mutations of each SOCS gene based on cBioPortal.

### STRING

STRING (https://string-db.org/) is a comprehensive web portal that integrates and scores all publicly available sources of protein–protein interaction (PPI) information and complements these with computational predictions (Szklarczyk et al., 2019). We assessed the interactions of eight SOCS family members by conducting a PPI network analysis based on this comprehensive web resource.

### GeneMANIA

GeneMANIA (http://genemania.org/), a flexible web interface, can generate hypotheses about gene function and analyze gene lists (Warde-Farley et al., 2010). GeneMANIA can identify other genes related to a set of input genes and predict the function of input genes. We explored the functions of eight SOCS family members and the potential signaling pathways that they may contribute to by GeneMANIA.

### Functional Enrichment Analysis

We found other genes related to SOCS family members by GeneMANIA and obtained the Entrez Gene ID for SOCS family members and related genes by “org.Hs.eg.db” R package. Additionally, we performed the Gene Ontology (GO) enrichment analysis and Kyoto Encyclopedia of Genes and Genomes (KEGG) pathway enrichment analysis using the “clusterProfiler” R package. Finally, the results were visualized by “enrichplot” and “ggplot2” R packages. The GO enrichment analysis consisted of biological processes (BP), cellular components (CC), and molecular function (MF).

### TRRUST

TRRUST (https://www.grnpedia.org/trrust/) contains 8,444 regulatory relationships for 800 transcription factors (TFs) in humans (Han et al., 2018). We evaluated the potential TF targets of these SOCS family members using TRRUST.

### TIMER

TIMER (https://cistrome.shinyapps.io/timer/) is an interactive and flexible web portal that can provide systematical analysis of immune infiltrates of most cancer types (Li et al., 2017). We assessed the relationship between SOCS family members and immune infiltrates in KIRC by the “gene” module on the TIMER database.

### Cell Culture and Treatments

Human KIRC cell line 786-O was purchased from the Shanghai Institute of Cell Biology. 786-O was cultured in RPMI-1640 medium supplemented with 10% fetal bovine serum (Gibco, United States) and 1% penicillin/streptomycin (Gibco, United States). 786-O was grown in a humidified atmosphere of 5% CO_2_ at 37°C.

### Cell Transfection

To construct SOCS3 overexpression plasmids, human SOCS3 complementary DNA was synthesized and cloned into pCDNA3.1 vectors by Tsingke Biotechnology (Beijing, China). The empty plasmids served as the negative control. The 786-O cell line was transfected by jetPRIME (Polyplus-transfection, France) according to the manufacturer's instructions.

### Cell Proliferation, Migration, and Invasion Assays

A cell counting kit (CCK-8, Yeasen, China) was used for cell viability detection. In brief, cells were seeded in a 96-well plate at a density of 3,000 cells per well. Ten-microliter CCK-8 reagent was added to each well at 0, 24, 48, 72, and 96 h. Then, the absorbance was calculated at 450 nm *via* an automatic enzyme-linked immune detector after 2-h incubation.

The transwell chambers (8-μm pore size, Corning) without Matrigel (BD Science, United States) or with Matrigel were used for cell migration assays or invasion assays, respectively. In brief, 4 × 10^4^ 786-O cells were suspended in a 200-μl RPMI-1640 medium and plated in the top chamber. A total of 750 μl of RPMI-1640 medium supplemented with 20% fetal bovine serum was added into the lower chambers. After incubation for 16 h (migration assays) and 48 h (invasion assays), 786-O cells plated in the top chamber were scraped with cotton swabs, stained with crystal violet for photographing and counting.

### Western Blot Analysis

The cell was lysed using radio-immunoprecipitation assay lysis buffer (Yeasen, China). The protein concentration was calculated by BCA Protein Quantification Kit (Yeasen, China). Then, total protein was separated by electrophoresis using 12% sodium dodecyl sulfate–polyacrylamide gel electrophoresis, electro-transferred onto polyvinylidene fluoride membranes (Millipore, Germany), and incubated with primary antibodies overnight. The membranes were incubated with specific horseradish peroxidase-conjugated secondary antibodies for approximately 1 h at room temperature. The images were acquired using FluorChem System. Antibodies used included anti-SOCS3 (1:2,000, Proteintech), β-actin (1:2,000, Proteintech), and horseradish peroxidase-conjugated secondary goat anti-mouse (1:5,000, Biosharp).

### Survival Analysis

To evaluate the overall survival (OS) of different expression levels of eight SOCS genes, we performed Kaplan–Meier analysis by the “limma” and “survival” R packages. Briefly, we determined high and low SOCS expression groups by utilizing the “limma” package and then integrated the clinical data with RNA-seq data downloaded from TCGA database. Patients with KIRC (*n* = 537) were divided into different subgroups based on varying expression levels of each SOCS gene. Finally, Kaplan–Meier analysis was performed to assess OS of different subgroups.

### Construction of Kidney Renal Clear Cell Carcinoma Prognostic Index for Suppressor of Cytokine Signaling Genes

Univariate Cox regression analysis was carried out to assess the predictive value of SOCS family members. Seven SOCS genes were verified as predictive factors for KIRC. We then performed the multivariate Cox regression analysis to determine the regression coefficients of seven SOCS genes and created a KIRC prognostic index (KIRCPI) for predicting the prognosis of patients with KIRC. KIRCPI was calculated as follows:

$$\mathrm{KIRCPI =}e^{y}\mathrm{. y=}\sum \left[\left(x_{i}\mathrm{-xi}\right)*\beta_{i}\right]$$

where x_i_ is the expression level of gene i and xi is the average value of expression of gene i, whereas β_i_ is its regression coefficient. We divided each patient with KIRC into high-risk or low-risk groups according to their KIRCPI. The Kaplan–Meier curve with log-rank test was performed to compare the survival difference between different subgroups. The receiver operating characteristic (ROC) curve was used to evaluate the predictive value of KIRCPI in patients with KIRC. Finally, we assessed the prognostic value of KIRCPI in patients with KIRC under similar clinicopathologic characteristics and performed the ROC curve to evaluate the predictive accuracy of KIRCPI.

### Statistical Analysis

The continuous variables were compared using Student's *t*-test, whereas the chi-square test or Fisher's exact test was carried out for categorical variables. The Wilcoxon rank-sum test was carried out to compare the differences between the two groups. Kaplan–Meier curves with the log-rank test were used to evaluate OS of different subgroups. Univariate and multivariate Cox regression analyses were used to determine the independent prognostic gene signature. All statistical analyses were performed using IBM SPSS Statistics 22.0 and R software 4.0.0. A value of *p* < 0.05 was considered statistically significant for all statistical analyses.

## Results

### Aberrant Expression of Suppressor of Cytokine Signaling Family Members in Kidney Renal Clear Cell Carcinoma

**FIGURE 1:**
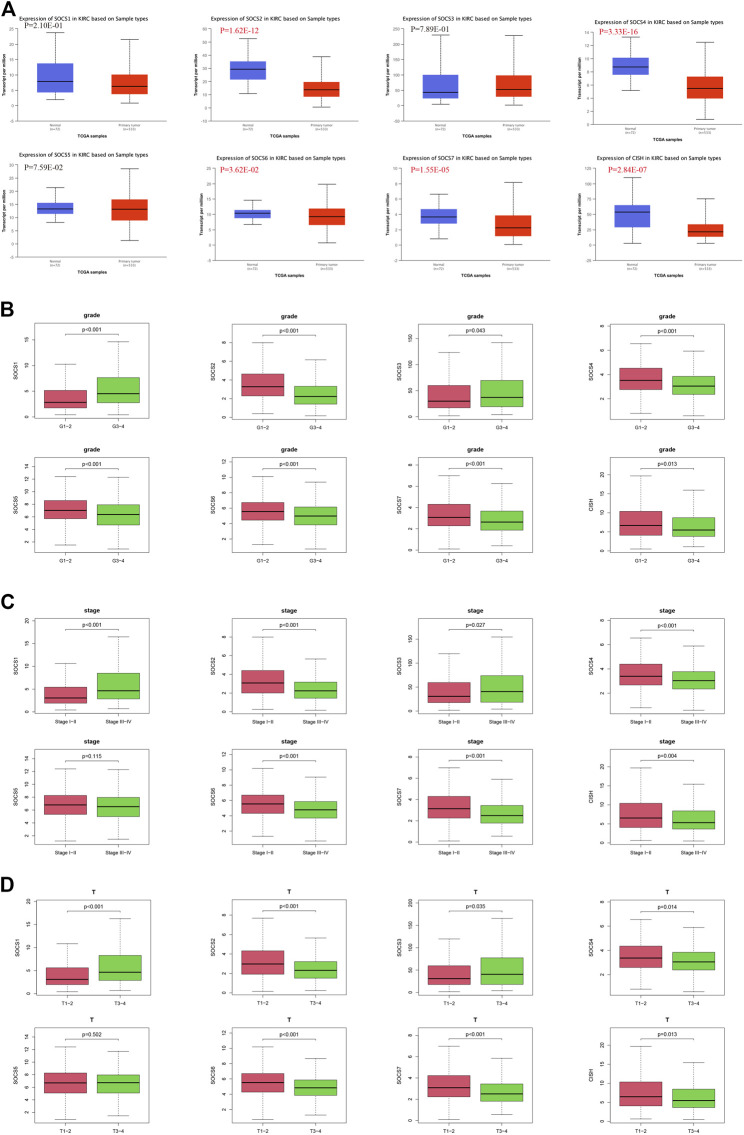
Expression levels of SOCS family members in KIRC and correlation with different clinicopathological features. **(A)** Expression levels of SOCS family members in KIRC were determined by UALCAN. **(B)** Correlation between expression levels of each SOCS family member and histological grade. **(C)** Correlation between expression levels of SOCS family members and pathological stage. **(D)** Correlation between expression levels of SOCS family members and T stage.

The expression levels of SOCS genes were retrieved from the UALCAN database. We analyzed and compared the expression of SOCS family members between KIRC and normal renal tissues. As shown in Figure 1A, among the eight SOCS family members, the expression levels of *SOCS2* (*P* = 1.62E-12), *SOCS4* (*P* = 3.33E-16), *SOCS6* (*P* = 3.62E-02), *SOCS7* (*P* = 1.55E-05), and *CISH* (*P* = 2.84E-07) were significantly suppressed in KIRC *vs*. normal renal tissues, whereas no differential expression was observed for *SOCS1*, *SOCS3*, and *SOCS5*.

**FIGURE 2:**
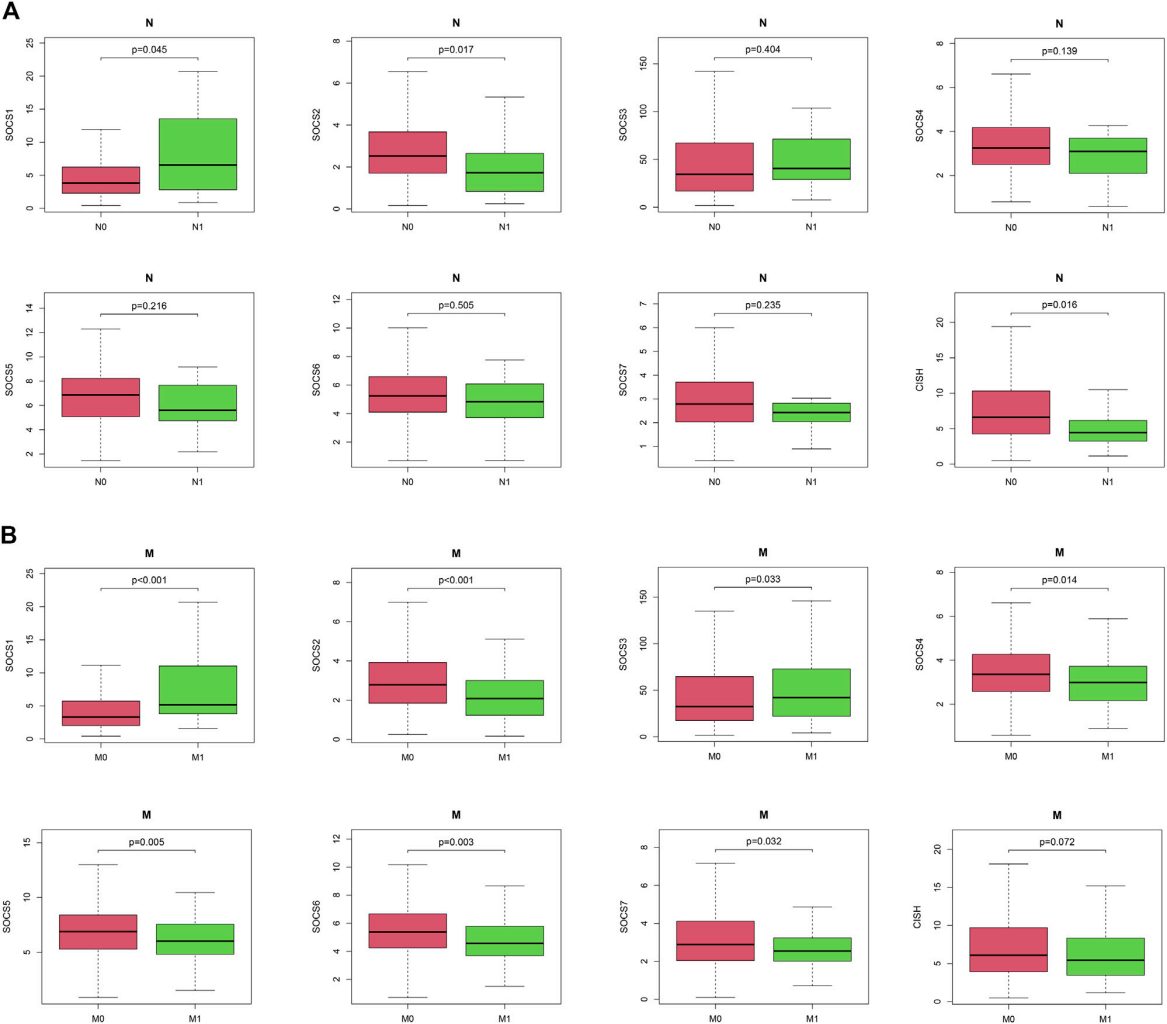
Correlation between expression levels of SOCS genes and different clinicopathological features in patients with KIRC. **(A)** Correlation between expression level of each SOCS family member and N stage. **(B)** Correlation between expression level of SOCS family members and M stage.

We next embarked on an integrated search for the correlation between the transcriptional levels of SOCS genes and different clinicopathological features in patients with KIRC. We demonstrated *SOCS1* (*p* < 0.001) and *SOCS3* (*P* = 0.043) were upregulated as the histological grade increased (Figure 1B). The expression levels of other SOCS genes, including *SOCS2*, *SOCS4*, *SOCS5*, *SOCS6*, *SOCS7*, and *CISH*, were lower in G3 and G4 as compared with those of G1 and G2. As the KIRC staging was elevated, *SOCS1* and *SOCS3* had higher expression levels, whereas *SOCS2*, *SOCS4*, *SOCS6*, *SOCS7*, and *CISH* had lower expression levels (Figure 1C). Specifically, in comparison with that of T3 and T4, T1 and T2 showed downregulation of *SOCS1* and *SOCS3* and upregulation of *SOCS2*, *SOCS4*, *SOCS6*, *SOCS7*, and *CISH* (Figure 1D). Among the eight SOCS genes, *SOCS1*, *SOCS2*, and *CISH* were associated with lymphatic metastasis (Figure 2A), and *SOCS1-7* was associated with distant metastasis in KIRC (Figure 2B). High expression of *SOCS1* and low expression of *SOCS2* and *CISH* increased the likelihood of lymphatic metastasis. High expression of *SOCS1* and *SOCS3* and low expression of *SOCS2*, *SOCS4*, *SOCS5*, *SOCS6*, and *SOCS7* were significantly correlated with a higher likelihood of distant metastasis of KIRC. These results demonstrate that SOCS family members may act pivotal parts in tumorigenesis of KIRC and act as prognostic biomarkers.

### Overexpression of SOCS3 Inhibited Proliferation, Migration, and Invasion *In Vitro*

**FIGURE 3:**
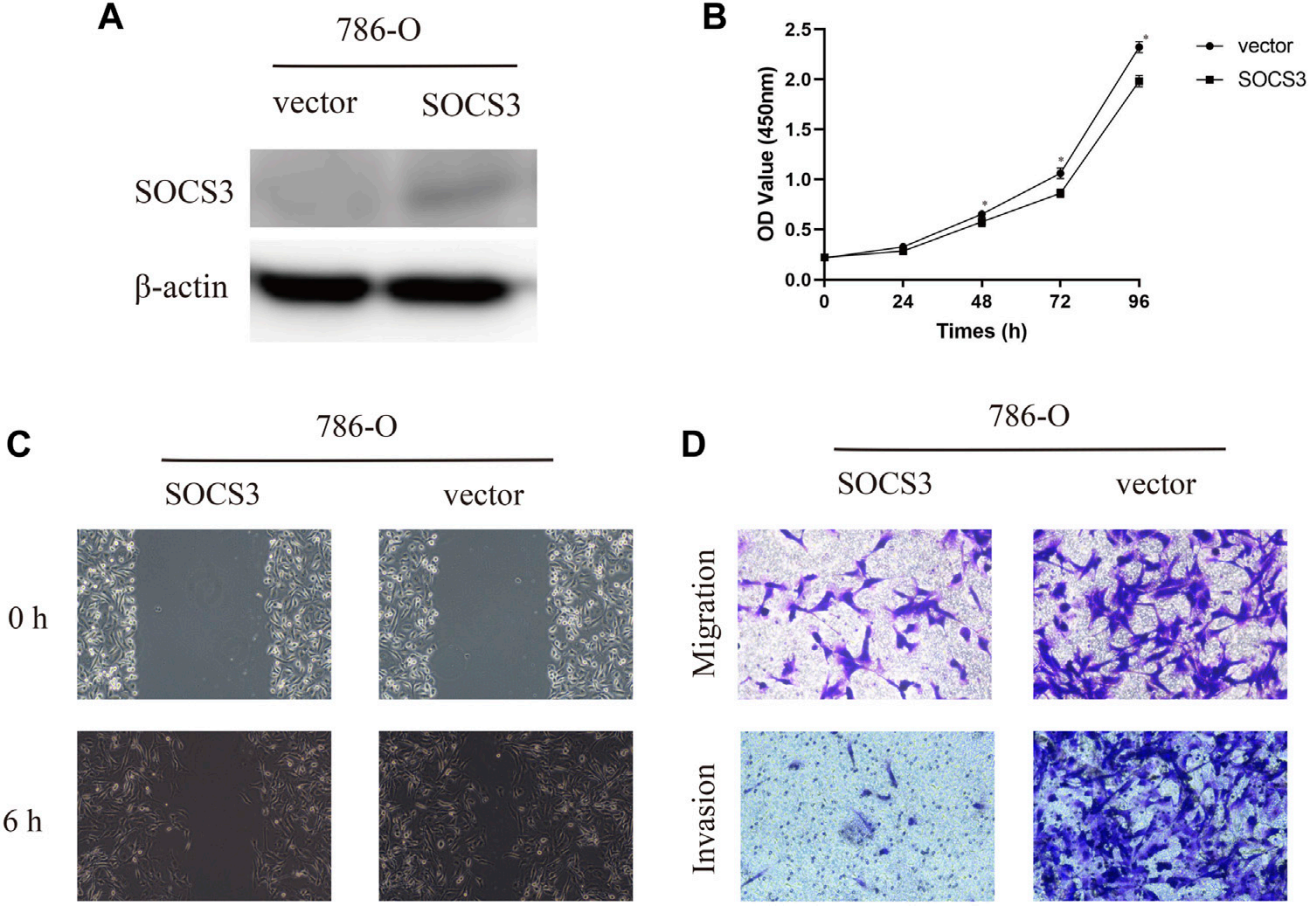
Overexpression of SOCS3 inhibited proliferation, migration, and invasion. **(A)** Western blot revealed that SOCS3 overexpression plasmids lead to an upregulation of SOCS3. **(B)** SOCS3 inhibited cell proliferation by CCK-8 assay. **(C)** Wound healing assay showed that overexpression of SOCS3 inhibited a closing of scratch wounds. **(D)** Overexpression of SOCS3 inhibited cell migration and invasion.

To further evaluate the function of SOCS3 in KIRC, we transfected SOCS3 overexpression plasmids into a 786-O cell. Western blot analysis revealed that SOCS3 overexpression plasmids lead to an apparent upregulation of SOCS3 (Figure 3A). The cck-8 assay revealed that overexpression of SOCS3 inhibited cell proliferation (Figure 3B). Wound healing assay revealed that overexpression of SOCS3 suppressed cell migration in 786-O (Figure 3C). Consistently, overexpression of SOCS3 also inhibited migration and invasion of the 786-O cell (Figure 3D).

### Genetic Alterations, Co-Expression, Protein-Protein Interaction Network, and Functional Analysis of Suppressor of Cytokine Signaling in Patients With Kidney Renal Clear Cell Carcinoma

**FIGURE 4:**
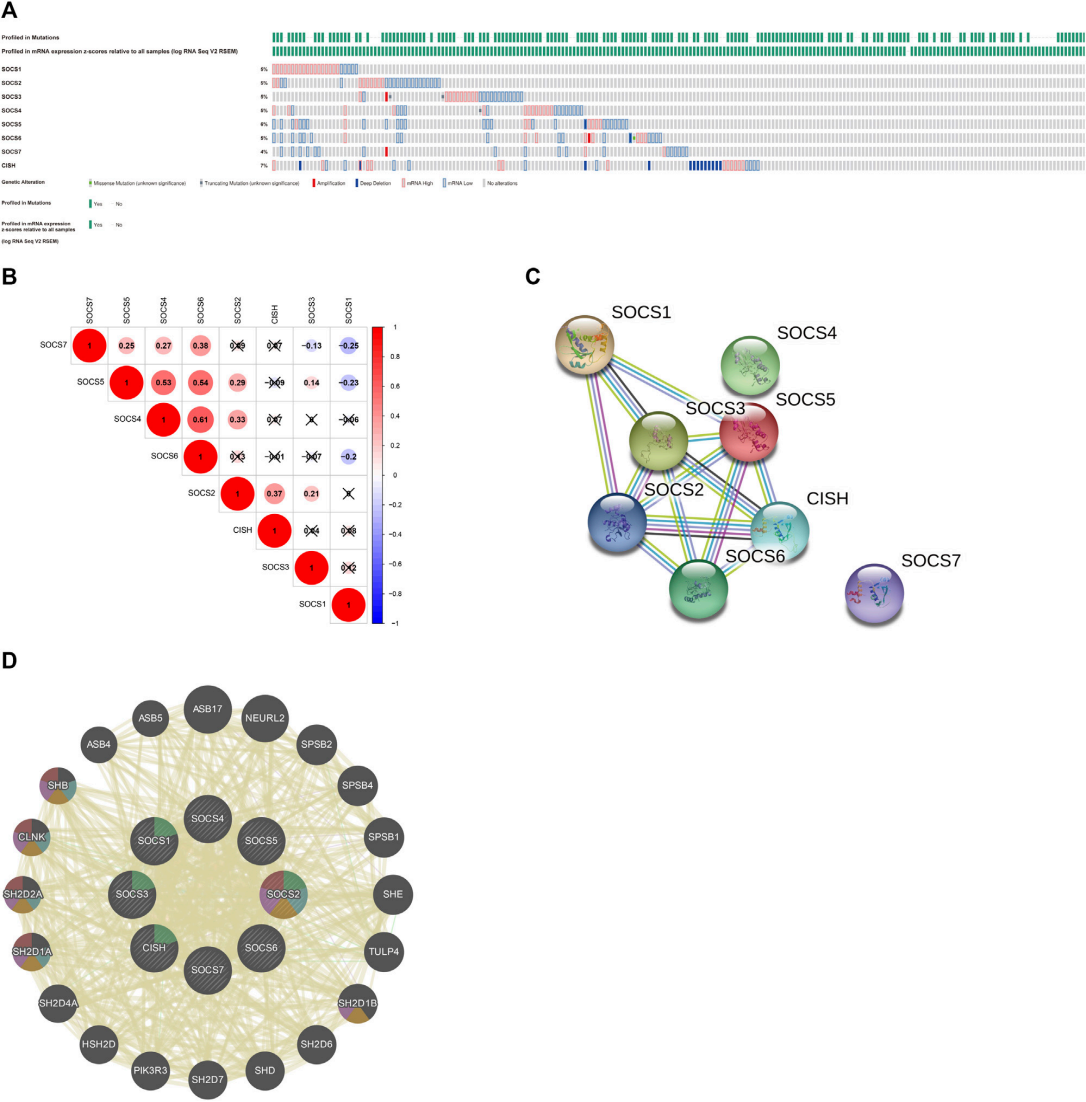
**(A)** Genetic alterations of different SOCS family members in KIRC. **(B)** Potential co-expression analysis indicated a significant correlation was observed among *SOCS4*, *SOCS5*, and *SOCS6*. **(C)** Protein–protein interaction network demonstrated *SOCS2*, *SOCS3*, and *SOCS5* were possibly hub genes among these SOCS family members. **(D)** SOCS family members were predominantly related to protein binding, SH3/SH2 adapter activity, signaling adapter activity, and JAK/STAT cascade.

We first assessed the genetic alterations in different SOCS family members in KIRC using the cBioportal for Cancer Genomics database. As a result, upregulation and downregulation of the RNA levels were the most common genetic alterations in seven of eight SOCS family members, except *CISH*; deep deletion was the most common genetic alteration in the *CISH* gene (Figure 4A). *SOCS1-7* and *CISH* were altered in 5, 5, 5, 5, 6, 5, 4, and 7% of the KIRC tissues, respectively. We then assessed the potential co-expression among eight SOCS family members. As shown in Figures 4A, B, a significant correlation was observed among *SOCS4*, *SOCS5*, and *SOCS6*. *SOCS7* was positively correlated to *SOCS4*, *SOCS5*, and *SOCS6* and negatively correlated to *SOCS1* and *SOCS3*. *SOCS1* was negatively associated with *SOCS5*, *SOCS6*, and *SOCS7*. A low to moderate correlation was observed among *SOCS2* and *CISH*, *SOCS3*, *SOCS4*, and *SOCS5*. Next, we performed a PPI network analysis to evaluate the correlation among these SOCS family members using STRING. We hypothesized that *SOCS2*, *SOCS3*, and *SOCS5* were possibly the hub genes among these SOCS family members (Figure 4C). However, there was no evidence to support the potential interactions between *SOCS4* and *SOCS7* and other SOCS family members. Additionally, we explored the functions of 8 SOCS family members and potential signaling pathways that they may be involved in by GeneMANIA. As shown in Figure 4D, these SOCS family members were predominantly related to protein binding, SH3/SH2 adapter activity, signaling adapter activity, and JAK/STAT cascade.

### Potential Functions of Suppressor of Cytokine Signaling Family Members in Patients With Kidney Renal Clear Cell Carcinoma

**FIGURE 5:**
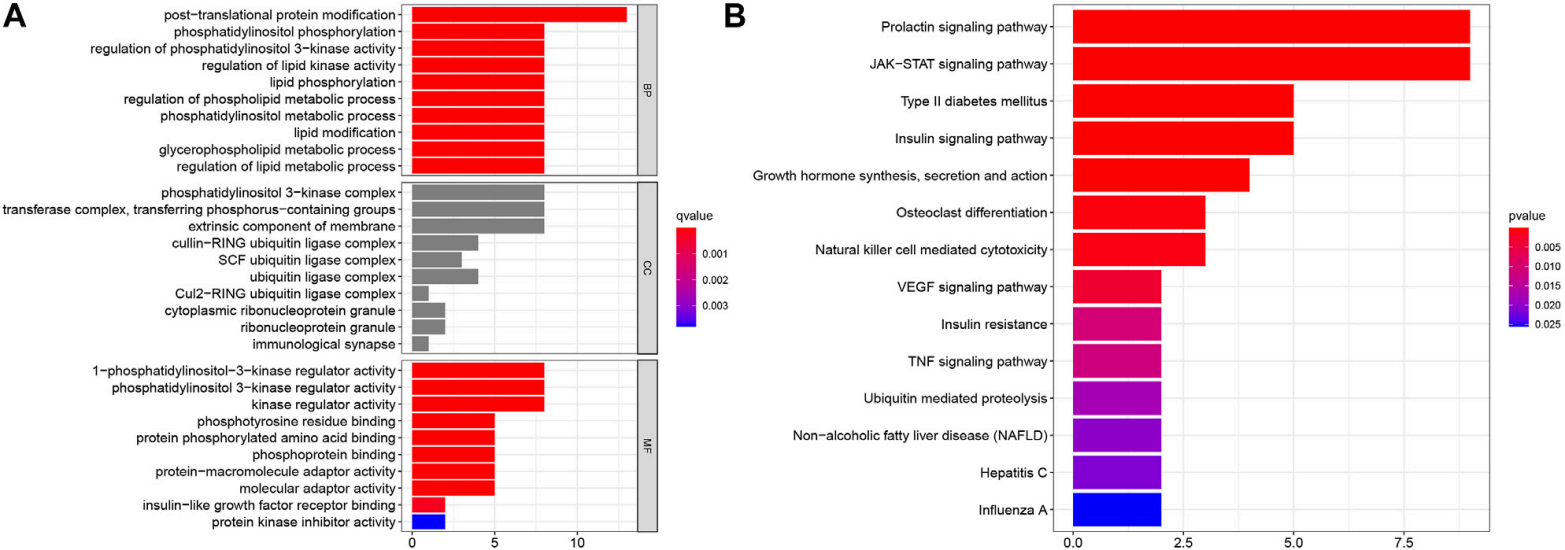
Results of GO enrichment analysis and KEGG pathway enrichment analysis. **(A)** Results of GO enrichment analysis in CC, BP, and MF categories. **(B)** Results of KEGG pathway enrichment analysis.

To evaluate the potential functions of SOCS genes and neighboring genes, we conducted GO enrichment analysis and KEGG pathway enrichment analysis. As shown in Figure 5A, in the BP category, SOCS genes and related genes were highly enriched in posttranslational protein modifications, phosphatidylinositol phosphorylation, regulation of phosphatidylinositol 3-kinase activity, regulation of lipid kinase activity, lipid phosphorylation, regulation of phospholipid metabolic process, phosphatidylinositol metabolic process, lipid modification, glycerophospholipid metabolic process, and regulation of lipid metabolic process. In the CC category, the phosphatidylinositol 3-kinase complex, transferase complex, extrinsic component of membrane, cullin-RING ubiquitin ligase complex, SCF ubiquitin ligase complex, and ubiquitin ligase complex were markedly related to the tumorigenesis of KIRC. In the MF category, 1-phosphatidylinositol-3-kinase regulator activity, phosphatidylinositol 3-kinase regulator activity, kinase regulator activity, phosphotyrosine residue binding, protein phosphorylated amino acid binding, phosphoprotein binding, protein-macromolecule adaptor activity, molecular adaptor activity, insulin-like growth factor receptor binding, and protein kinase inhibitor activity were highly enriched in tumorigenesis and progression in KIRC. As shown in Figure 5B, in the KEGG pathway enrichment analysis, SOCS genes and related genes were highly enriched in the prolactin signaling pathway, JAK/STAT signaling pathway, type II diabetes mellitus, insulin signaling pathway, growth hormone synthesis, secretion and action, osteoclast differentiation, natural killer cell-mediated cytotoxicity, and vascular endothelial growth factor signaling pathway.

### Transcription Factor Targets of Suppressor of Cytokine Signaling Family Members

We evaluated the potential TF targets of these SOCS family members using TRRUST. As shown in Table 2, STAT3, STAT6, and IRF1 may act as key TFs related to the regulation of SOCS. STAT3 could be the TF that is associated with the regulation of *SOCS1*, *SOCS3*, and *CISH*. STAT6 may function as a TF for *SOCS1* and *CISH*. IRF1 may serve as a TF for regulating *SOCS1* and *SOCS2*.

**TABLE 2:** Potential transcription factor targets of SOCS family members.

Key TF	Description	Of overlapped genes	*p*-Value	FDR^a^
STAT3	signal transducer and activator of transcription 3 (acute-phase response factor)	CISH, SOCS3, SOCS1	2.27E-05	6.82E-05
STAT6	signal transducer and activator of transcription 6, interleukin-4 induced	CISH, SOCS1	9.84E-05	0.000148
IRF1	interferon regulatory factor	SOCS1, SOCS2	0.000198	0.000198

a False discovery rate (FDR).

### Immune Infiltration of SOCS Family Members in Patients With Kidney Renal Clear Cell Carcinoma

**FIGURE 6:**
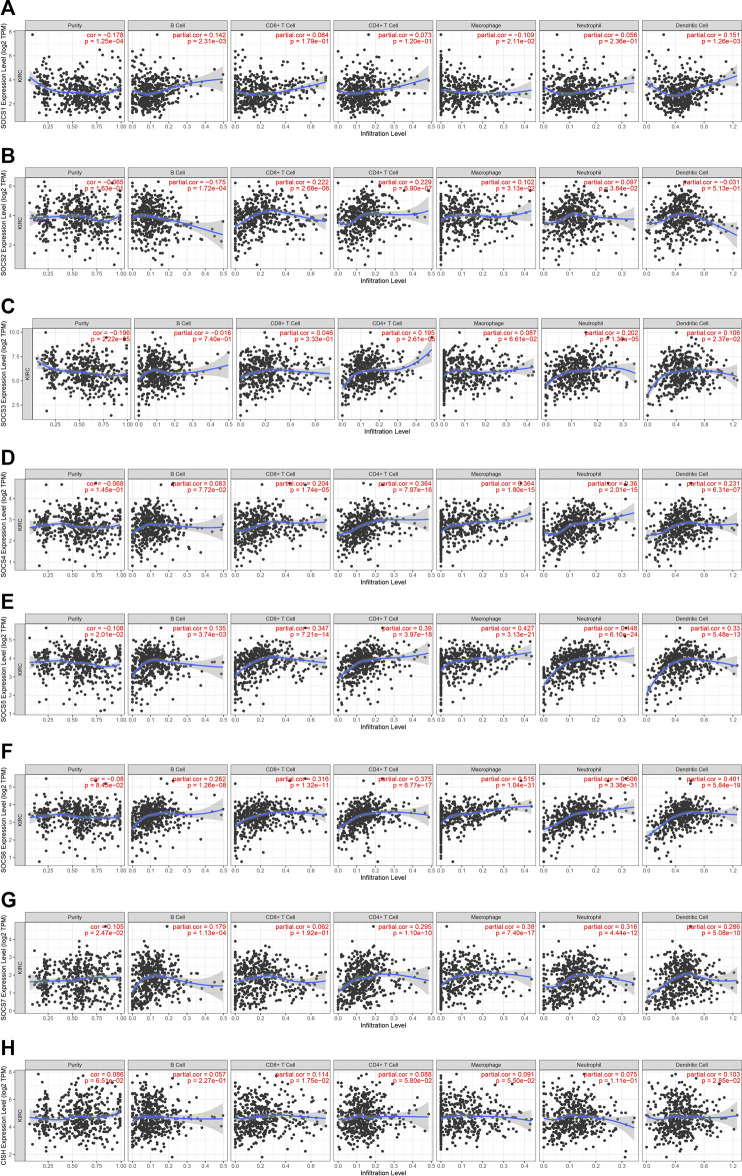
Correlation of SOCS expression with immune infiltration level in KIRC. **(A)** SOCS1 expression was positively associated with infiltration of dendritic and B cells. **(B)** SOCS2 expression was positively associated with infiltration of CD8^+^ T cells, CD4^+^ T cells, macrophages, and neutrophils and negatively associated with infiltration of B cells. **(C)** SOCS3 expression was positively associated with infiltration of CD4^+^ T cells, neutrophils, and dendritic cells. **(D)** SOCS4 expression was positively associated with infiltration of CD8^+^ T cells, CD4^+^ T cells, neutrophils, dendritic cells, and macrophages. **(E, F)** SOCS5 and SOCS6 expression were positively associated with infiltration of all six types of immune cells. **(G)** SOCS7 expression was positively associated with infiltration of B cells, CD4^+^ T cells, neutrophils, dendritic cells, and macrophages. **(H)** CISH expression was positively associated with infiltration of CD8^+^ T cells and dendritic cells.

Because SOCS family members were enriched in NK cell-mediated cytotoxicity, we hypothesized that SOCS genes may participate in the immune infiltrates of KIRC. We explored the potential association between SOCS family members and immune infiltrates by TIMER. We found that the expression of *SOCS1* was positively associated with the infiltration of dendritic (cor = 0.151, *P* = 1.26e-3) and B cells (cor = 0.142, *P* = 2.31e-3), whereas the expression of *SOCS1* was negatively associated with the infiltration of macrophages (cor = −0.109, *P* = 2.11e-2) (Figure 6A). A positive association was also found between *SOCS2* and infiltration of CD8^+^ T cells (cor = 0.222, *P* = 2.68e-6), CD4^+^ T cells (cor = 0.229, *P* = 2.90e-7), macrophages (cor = 0.102, *P* = 3.13e-2), and neutrophils (cor = 0.097, *P* = 3.84e-2), whereas *SOCS2* expression was negatively associated with the infiltration of B cells (cor = −0.175, *P* = 1.72e-4) (Figure 6B). We found a positive association among the transcriptional expression levels of *SOCS3* and CD4^+^ T cells (cor = 0.195, *P* = 2.61e-5), neutrophils (cor = 0.202, *P* = 1.30e-5), and dendritic cells (cor = 0.106, *P* = 2.37e-2) (Figure 6C). The transcriptional levels of *SOCS4* were positively related to the infiltration of CD8^+^ T cells (cor = 0.204, *P* = 1.74e-5), CD4^+^ T cells (cor = 0.364, *P* = 7.97e-16), neutrophils (cor = 0.360, P = 2.01e-15), dendritic cells (cor = 0.231, *P* = 6.31e-7), and macrophages (cor = 0.364, *P* = 1.80e-15) (Figure 6D). Except for CD8^+^ T cells, the expression of *SOCS7* was positively linked with the five other types of cells, including B cells (cor = 0.179, *P* = 1.13e-4), CD4^+^ T cells (cor = 0.295, *P* = 1.10e-10), neutrophils (cor = 0.316, *P* = 4.44e-12), dendritic cells (cor = 0.286, *P* = 5.08e-10), and macrophages (cor = 0.380, *P* = 7.40e-17) (Figure 6G). There was a positive relationship between the transcriptional levels of *CISH* and CD8^+^ T cells (cor = 0.113, *P* = 1.75e-2) and dendritic cells (cor = 0.103, *P* = 2.85e-2) (Figure 6H). Additionally, we found that the expression levels of *SOCS5* and *SOCS6* were positively linked with the infiltration of B cells, CD8^+^ T cells, CD4^+^ T cells, neutrophils, dendritic cells, and macrophages (Figures 6E, F).

### Suppressor of Cytokine Signaling Family Members Associate With the Prognosis of Kidney Renal Clear Cell Carcinoma

**FIGURE 7:**
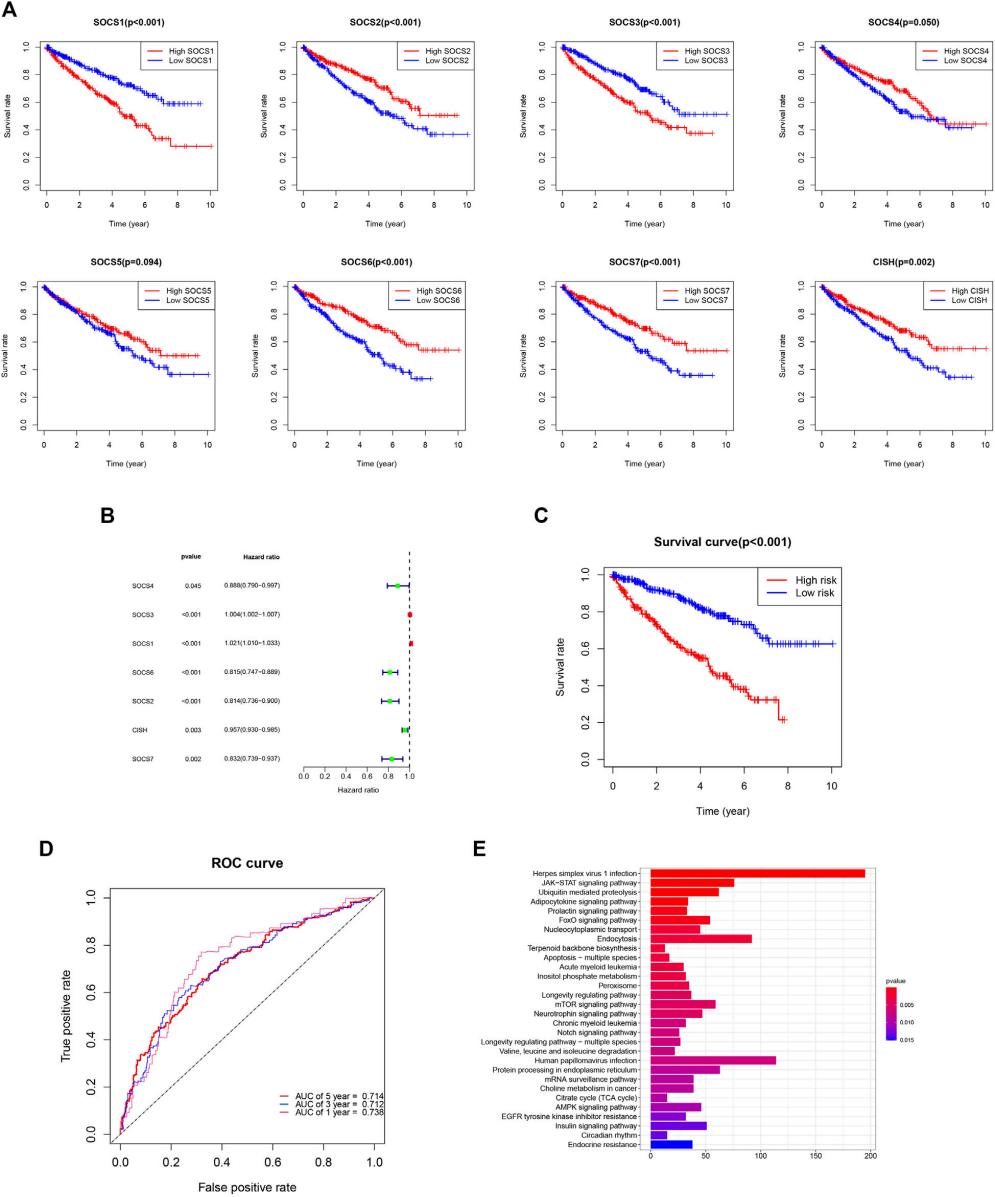
**(A)** Prognostic value of SOCS family members in overall survival curve. **(B)** Hazard ratio of SOCS family members in univariate Cox regression analysis. **(C)** Survival analysis demonstrated KIRC patients in high-risk group had shorter overall survival than those in low-risk group. **(D)** ROC curves for 1-, 3-, and 5-year survival prediction. **(E)** The results of KEGG pathway enrichment analysis.

We evaluated the prognostic value of SOCS genes and demonstrated that among the eight SOCS family members, *SOCS1*, *SOCS2*, *SOCS3*, *SOCS6*, *SOCS7*, and *CISH* were remarkably associated with OS in patients with KIRC (Figure 7A). KIRC patients with a higher *SOCS1* (*p* < 0.001) and *SOCS3* (*p* < 0.001) expression levels were related to worse OS. Decreased transcriptional levels of the other SOCS, including *SOCS2* (*p* < 0.001), *SOCS6* (*p* < 0.001), *SOCS7* (*p* < 0.001), and *CISH* (*P* = 0.002), were linked with shorter OS.

We next implemented the univariate Cox regression analysis to verify the prognostic value of SOCS genes. As expected, seven of eight SOCS family members, except *SOCS5*, were related to OS in patients with KIRC (Figure 7B). *SOCS1* and *SOCS3* were part of the risky genes with HR > 1, whereas *SOCS2*, *SOCS4*, *SOCS6*, *SOCS7*, and *CISH* were part of the protective genes with HR < 1. Multivariate Cox regression analysis was carried out to determine the regression coefficients of seven SOCS genes. *SOCS4*, *SOCS3*, *SOCS6*, *SOCS2*, and *CISH* were among KIRCPI, and the regression coefficients were acquired from the multivariate Cox regression model. KIRCPI was calculated as follows:

$$\begin{array}{l} \mathrm{KIRCPI =}e^{y}\mathrm{. y = 0}\mathrm{.228551 *}\left(\mathrm{SOCS4}\mathrm{- 1}\mathrm{.088096}\right)\mathrm{+ 0}\mathrm{.006333 *}\left(\mathrm{SOCS3}\mathrm{- 34}\mathrm{.77299}\right)\mathrm{- 0}\mathrm{.258678 *}\\ \left(\mathrm{SOCS6}\mathrm{- 1}\mathrm{.534994}\right)\mathrm{- 0}\mathrm{.229725 *}\left(\mathrm{SOCS2}\mathrm{- 1}\mathrm{.539131}\right)\mathrm{-0}\mathrm{.025342 *}\left(\mathrm{CISH}\mathrm{- 4}\mathrm{.98075}\right) \end{array}$$

Patients with KIRC in the high-risk group were associated with worse OS compared with those in the low-risk group (Figure 7C). The 5-year survival rate of the low-risk patients was 77.8% (95% CI: 0.715–0.847), whereas that of the high-risk group was 45.3% (95% CI: 0.3846–0.533). The ROC curve of 1, 3, and 5 years was generated to evaluate the predictive accuracy of KIRCPI (Figure 7D). We then conducted KEGG pathway enrichment analysis between two groups and revealed that herpes simplex virus 1 infection, JAK-STAT signaling pathway, and ubiquitin-mediated proteolysis were highly enriched (Figure 7E).

### Validation of the Kidney Renal Clear Cell Carcinoma Prognostic Index by International Cancer Genome Consortium Database

**FIGURE 8:**
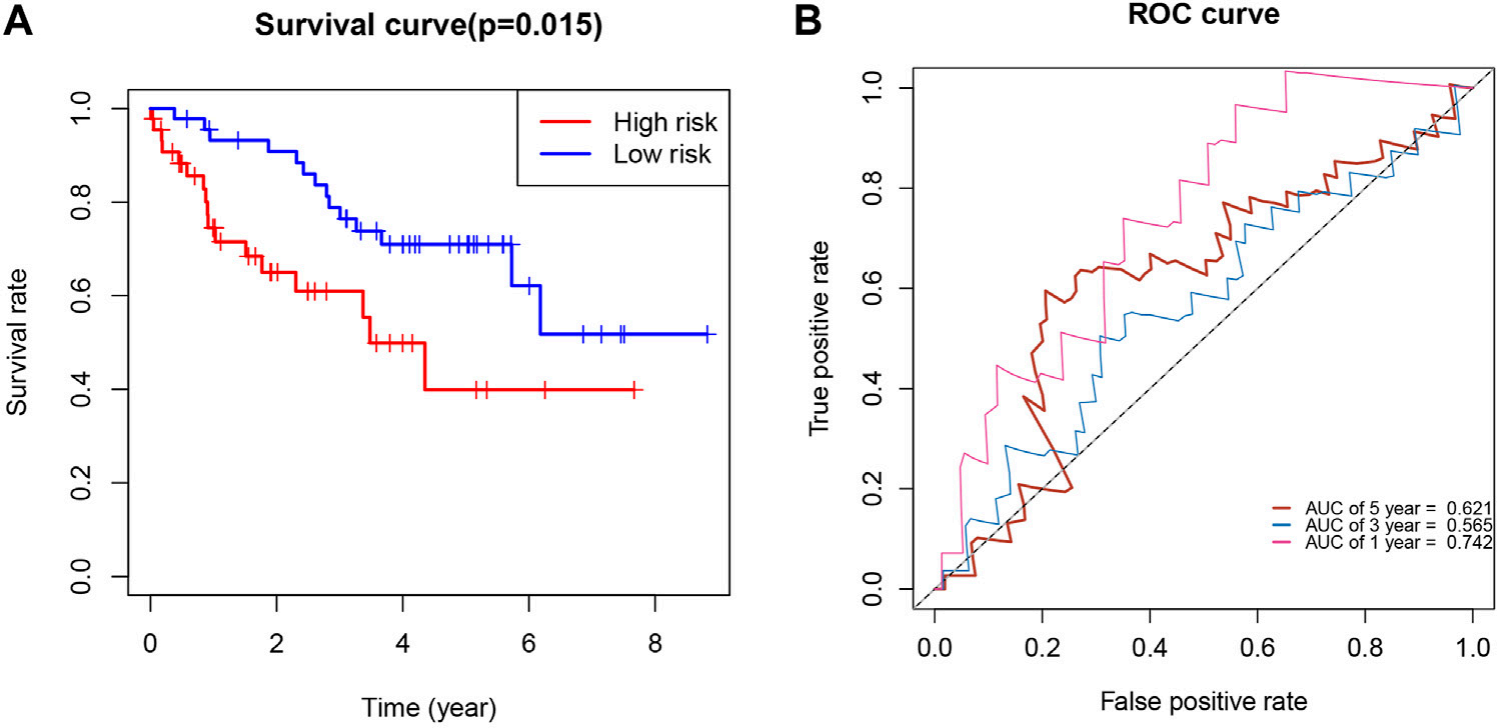
**(A)** Survival analysis demonstrated KIRC patients in high-risk group had shorter overall survival than those in low-risk gourp. **(B)** ROC curves for 1-, 3-, and 5-year survival prediction.

We used the International Cancer Genome Consortium cohort to further validate the prognostic value of the KIRCPI. We used the same formula as TCGA cohort to calculate the risk score of each patient and set the median score as the cutoff to divide the patients into high- and low-risk groups. The Kaplan–Meier curve, as expected, showed that patients in the high-risk group were associated with worse OS compared with those in the low-risk group (Figure 8A). The ROC curve of 1, 3, and 5 years was plotted to validate the prognostic accuracy (Figure 8B).

### Kidney Renal Clear Cell Carcinoma Prognostic Index Can Predict the Prognosis of Patients With Kidney Renal Clear Cell Carcinoma Under Similar Clinicopathological Characteristics

**FIGURE 9:**
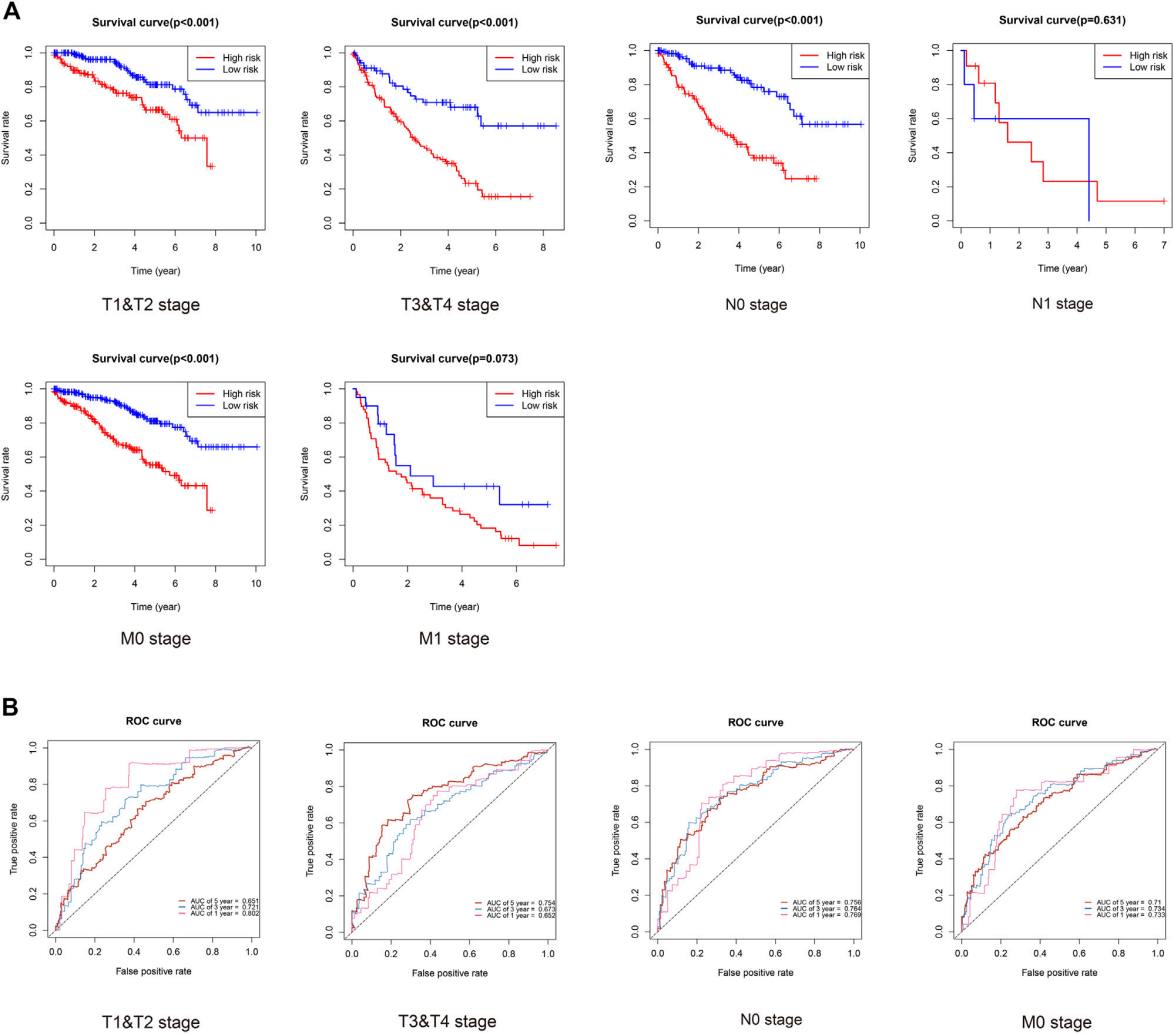
Prognosis of KIRC patients under same clinicopathologic characteristics and ROC curves for 1-, 3-, and 5-year survival prediction.

Finally, we evaluated the prognosis of patients with KIRC with similar clinicopathological characteristics. As expected, KIRCPI could be used to evaluate the prognosis of patients with KIRC with similar clinicopathologic characteristics. As shown in Figure 9A, patients with KIRC in the high-risk group were related to worse OS than low-risk group's patients, in T1 and T2, T3, and T4, and N0 or M0, whereas in N1 and M1 stages, no difference was found between the two groups. The ROC curve of 1, 3, and 5 years was generated to evaluate the predictive accuracy of KIRCPI (Figure 9B).

## Discussion

SOCS family members were initially identified to regulate the cellular responses to cytokines and growth factors and were found to be the key negative regulating factors in several signaling pathways, including the JAK/STAT pathway (Linossi and Nicholson, 2015). An increasing number of researchers have shown that SOCS genes act as important roles in tumorigenesis, progression, invasion, and angiogenesis in several cancers. Nevertheless, few studies have revealed the relationship between SOCS and KIRC, and specific biological functions of SOCS family genes in KIRC have not been illuminated.

In our study, we confirmed the antineoplastic function of *SOCS3 in vitro*. Overexpression of *SOCS3* inhibited cell proliferation, migration, and invasion *in vitro*. As one of the most key negative regulating factors of the JAK/STAT signaling pathway, *SOCS3* downregulated cancer progression in lung and pancreatic cancers, which are similar to our analysis *in vitro* (He et al., 2003; Lesina et al., 2011). However, our clinical data suggested that *SOCS3* might act as a risk gene during the progression of KIRC. In comparison with that of T3 and T4, *SOCS1* and *SOCS3* were downregulated, and *SOCS2*, *SOCS4*, *SOCS6*, *SOCS7*, and *CISH* were upregulated in T1 and T2. Moreover, *SOCS1*, *SOCS2*, and *CISH* were associated with lymphatic metastasis, and *SOCS1-7* was associated with distant metastasis in KIRC. These findings demonstrated that SOCS family members might act as significant roles in the KIRC progression, and *SOCS3* might act as a critical protein in the progression of KIRC. Puhr et al. (2009) found downregulation of *SOCS3* promoted prostate cancer cell death through activation of the proapoptotic caspase-3/caspase-7, caspase-8, and caspase-9. IL-6R antibody with interferon inhibited RCC growth *in vitro* and *in vivo via* suppressed SOCS3, indicating the critical function of *SOCS3* in the progression of KIRC (Oguro et al., 2013). Mice lacking *SOCS3* exhibited dramatic inflammatory phenotypes by activating specific cytokine receptors such as IFN-γ or IL-6 (Croker et al., 2003), (Roberts et al., 2001). Besides, exogenous SOCS3 reduced the production of inflammatory cytokines and attenuated liver apoptosis (Jo et al., 2005). It is becoming clear that the function of SOCS3 was often highly context-dependent. We supposed that SOCS3 functioned as an oncogene or an antioncogene depending on the cellular context. On the one hand, SOCS3 was one of the most key negative regulating factors of the JAK/STAT signaling pathway; on the other hand, SOCS3 inhibited immune infiltration of tumor microenvironment by reducing the production of inflammatory cytokines.

We then assessed the genetic alterations of the SOCS family of genes in KIRC. Among these SOCS genes, high and low mRNA levels were the most common genetic alterations. A significant correlation was observed among these SOCS family members, and these genes were predominantly related to SH3/SH2 adaptor activity, signaling adaptor activity, and JAK/STAT cascade. As tumorigenesis is the result of the complex interactions of multiple genes, factors, and signaling pathways, SOCS genes may play a synergistic role in this biological process. We conducted the GO enrichment analysis and KEGG pathway enrichment analysis, and as expected, SOCS genes and related genes were enriched in posttranslational protein modifications, kinase regulator activity, and JAK/STAT pathway. The JAK/STAT pathway regulates embryonic development, stem cell maintenance, hematopoiesis, and inflammatory response (Thomas et al., 2015). Aberrant activated JAK/STAT signaling pathway plays a critical role in multiple cancers (Chen E et al., 2012). The JAK/STAT pathway is aberrantly activated in KIRC. Horiguchi et al. found that the expression levels of JAK3, a significant kinase that regulated the JAK/STAT pathway, were upregulated in KIRC tissues (Liang et al., 2020a). Lue et al. (2015) found that a combination of the Src and JAK/STAT inhibitors could promote tumor inhibition in RCC, indicating that the JAK/STAT pathway may function as a potential therapeutic target. As key negative regulating factors of the JAK/STAT pathway, the SOCS family of genes may act as an important role in KIRC treatment in the future.

We then evaluated the potential TFs that may regulate these SOCS family members. We have demonstrated that STAT3, STAT6, and IRF1 act as important TFs that can regulate these SOCS genes. STAT3 plays a significant role in tumor progression both in a tumor cell-intrinsic manner and through its ability to modulate the activity of the surrounding cell milieu (Huynh et al., 2019). STAT3 can accelerate the proliferation of gastric cancer by mediating the upregulation of vascular endothelial growth factor expression (Wu et al., 2016). STAT3 knockout can enhance the inhibitory effect of anthracycline-based chemotherapies on tumor cells (Yang et al., 2015). STAT6 acts as an important TF that takes part in the cell cycle, cell growth, and apoptosis. DiScala et al. (2020) found that loss of STAT6 results in trastuzumab resistance in HER2+ breast cancer cells. IRF1 functions in immune response, DNA damage, and DNA repair. IRF1 is downregulated in colorectal cancer and suppresses cell proliferation, migration, and metastasis (Hong et al., 2019). Moreover, in RCC, IRF1 can inhibit Ki-67 gene transcription by interfering with Sp1 activation (Chen F et al., 2012).

We evaluated the relationship between SOCS genes and immune infiltrates and found that the immune infiltrates of B cells, CD8^+^ T cells, CD4^+^ T cells, macrophages, neutrophils, and dendritic cells were significantly associated with the expression levels of SOCS genes. Finally, we assessed the prognostic value of differentially expressed SOCS and found that *SOCS1* and *SOCS3* are risk genes, whereas *SOCS2*, *SOCS4*, *SOCS6*, *SOCS7*, and *CISH* are part of the protective genes for patients with KIRC. We have created a KIRCPI for predicting the prognosis of patients with KIRC.

KIRCPI = e^y^. y = 0.228551 * (*SOCS4* - 1.088096) + 0.006333 * (*SOCS3* - 34.77299) - 0.258678 * (*SOCS6* - 1.534994) - 0.229725 * (*SOCS2* - 1.539131) -0.025342 * (*CISH* - 4.98075).

We found that KIRCPI can predict the prognosis of patients with KIRC under similar T1 and T2, T3, and T4, and N0 or M0 stages.

This study has several limitations. In our manuscript, we only speculated that SOCS3 contained multiple functions during KIRC progression and functioned as an oncogene or an antioncogene depending on the cellular context. However, it is still remained to be proven the potential mechanism and the practical role of SOCS3 during the progression of KIRC. Besides, all the samples used to establish the signature were retrospective samples; therefore, validation by prospective samples is necessary. In addition, as a previous study verified that aberrant expression of SOCS family members was observed in blood samples (Martínez-Baños et al., 2017), we believe our prognostic index contains potential usefulness in blood samples, and more research would be carried out.

In conclusion, we have assessed the transcriptional levels of SOCS family members and determined their correlation with the clinicopathological features in KIRC. SOCS family members have important value for predicting OS in patients with KIRC. Although our result indicated KIRCPI could not predict the prognosis of patients with KIRC under N1 or M1 stages, we speculate that this might be due to the small sample size of N1 stage and M1 stage and thus believe patients at any tumor–nodes–metastases stage might benefit from our prognostic index.

## Data Availability

The datasets presented in this study can be found in online repositories. The names of the repository/repositories and accession number(s) can be found in the article/Supplementary Material.
